# Relationship between insertion/deletion (indel) frequency of proteins and essentiality

**DOI:** 10.1186/1471-2105-8-227

**Published:** 2007-06-28

**Authors:** Simon K Chan, Michael Hsing, Fereydoun Hormozdiari, Artem Cherkasov

**Affiliations:** 1 CIHR/MSFHR Strategic Training Program in Bioinformatics, Canada's Michael Smith Genome Sciences Centre, 570 West 7th Ave – Suite 100, Vancouver, BC, V5Z 4S6, Canada; 2 Bioinformatics Graduate Program, University of British Columbia, 570 West 7th Ave – Suite 100, Vancouver, BC, V5Z 4S6, Canada; 3 School of Computing Science, Simon Fraser University, 8888 University Drive, Burnaby, BC, V5A 1S6, Canada; 4 Division of Infectious Diseases, Faculty of Medicine, University of British Columbia, 2733 Heather Street, Vancouver, BC, V5Z 3J5, Canada

## Abstract

**Background:**

In a previous study, we demonstrated that some essential proteins from pathogenic organisms contained sizable insertions/deletions (indels) when aligned to human proteins of high sequence similarity. Such indels may provide sufficient spatial differences between the pathogenic protein and human proteins to allow for selective targeting. In one example, an indel difference was targeted via large scale in-silico screening. This resulted in selective antibodies and small compounds which were capable of binding to the deletion-bearing essential pathogen protein without any cross-reactivity to the highly similar human protein. The objective of the current study was to investigate whether indels were found more frequently in essential than non-essential proteins.

**Results:**

We have investigated three species, *Bacillus subtilis, Escherichia coli*, and *Saccharomyces cerevisiae*, for which high-quality protein essentiality data is available. Using these data, we demonstrated with t-test calculations that the mean indel frequencies in essential proteins were greater than that of non-essential proteins in the three proteomes. The abundance of indels in both types of proteins was also shown to be accurately modeled by the Weibull distribution. However, Receiver Operator Characteristic (ROC) curves showed that indel frequencies alone could not be used as a marker to accurately discriminate between essential and non-essential proteins in the three proteomes. Finally, we analyzed the protein interaction data available for *S. cerevisiae *and observed that indel-bearing proteins were involved in more interactions and had greater betweenness values within Protein Interaction Networks (PINs).

**Conclusion:**

Overall, our findings demonstrated that indels were not randomly distributed across the studied proteomes and were likely to occur more often in essential proteins and those that were highly connected, indicating a possible role of sequence insertions and deletions in the regulation and modification of protein-protein interactions. Such observations will provide new insights into indel-based drug design using bioinformatics and cheminformatics tools.

## Background

Essential genes encode products that are required for the viability of an organism. There are two major reasons why there is considerable interest in determining the set of essential genes in an organism. Firstly, this will provide insights into the basic requirements needed to sustain a living cell. For example, the sequencing of the parasitic bacterium *Mycoplasma genitalium *[1] and the subsequent studies to determine its essential genes [2,3] have provided a more in-depth understanding of what constitutes a 'minimum genome.' Secondly, essential proteins in pathogens can potentially be excellent drug targets [4,5], as interfering with the proper functioning of one will likely interfere with an important pathway in the pathogen, thus reducing its threat to the host. However, targeting such essential proteins in a pathogen has one major drawback: essential proteins are often conserved across species, thus a drug that targets an essential protein in a pathogen may also cross-react with a similar host protein [6]. To combat this problem, our laboratory has recently developed a strategy to target insertions/deletions (indels) that occur among the proteins of a pathogen and its human host. For example, *Leishmania donovani *is a protozoan parasite that infects and inactivates the macrophages of its human host [7]. The main structural difference between the essential elongation factor (EF-1 α) protein of *L. donovani *and that of its human host is a 12 amino acid deletion that occurs in the *L. donovani *sequence [7]. The 12 amino acid sequence corresponds to a hair pin loop that is present in the human protein, but absent in the *L. donovani *protein. Using computational chemistry and molecular docking, we were able to develop inhibitors that directly recognized the exposed region in the *L. donovani *protein without any cross-reactivity to the highly similar human host protein [6,8,9]. Interestingly, this deletion can potentially allow EF-1 α from *L. donovani *to gain an interaction, relative to human EF-1 α, and interact with human tyrosine phosphatase, which leads to inactivation of the host macrophage [7]. With these past studies, we showed that indels can offer enough structural differences to target specific pathogen essential proteins as well as allow them to acquire and/or modify the protein-protein interactions that they are involved in.

Recently, we performed a large scale survey for potentially targetable indels by aligning the complete proteomes of bacterial and protozoan pathogens to the complete human proteome [10]. Our results showed that sizable indels were found in approximately 5–10% of bacterial proteins and as much as 25% of protozoan proteins with respect to human proteins. A large number of those proteins with indels were identified as being essential to their respective pathogens. Therefore, in this current study, we set out to determine if the frequency of indels in essential proteins differed from that of non-essential proteins. Our hypothesis is that essential proteins will likely contain more indels due to the following two observations: firstly, protein domain profiles characterized in databases such as Pfam [11] showed that protein sequences of the same protein interaction domain contained a large amount of residue variations across multiple species, which implied that a single point mutation in a protein did not have a large impact on the function of protein interaction domains. Secondly, essential proteins undergo stronger selective pressure and thus accumulate point mutations at a slower rate than non-essential proteins [12,13]. Therefore, taking these two considerations together, we propose that formation of indels may be one method by which proteins, especially those that are essential, use to acquire new interaction sites and/or modify existing ones, and thus their interaction partners. For example, it is well known that PINs tend to be scale-free [14,15], in which the majority of the proteins in an interaction network have much fewer interactions than the few highly connected 'hub' proteins. Due to the greater number of interactions that they participate in, hubs tend to be essential proteins. These hubs can gain interactions in the network if a gene encoding one of its interacting partners duplicates. This process is known as preferential attachment [14,15]. If an indel were to occur in the interaction site of the duplicate copy of the gene, then the resulting protein may reflect this change through a change in the number of interaction partners.

To our knowledge, the body of work presented here is the first to investigate a possible relationship between indel frequency and essentiality. We chose three species that have complete global knockout data: *Bacillus subtilis, Escherichia coli*, and *Saccharomyces cerevisiae*. Specifically, the purpose of this study was to determine 1) whether the mean indel frequency of essential proteins differed from that of non-essential proteins 2) whether the Weibull distribution could accurately model the indel abundances in both types of proteins 3) whether the indel frequency of a protein could be used as a marker to predict whether or not a given protein was essential and 4) whether proteins with indels participated in more interactions than those that do not. We defined indels as insertions and deletions between proteins of high sequence similarity (at least 50%), regardless of their evolutionary relationship with one another (i.e. not just orthologs between species). This work could potentially locate similar situations to the *L. donovani *case described and thus further explore the methodology of targeting indels of specific pathogen proteins without cross-reactivity to human host proteins.

## Results and discussion

### Query and subject species analyzed

To test whether the indel frequency of a protein is related to essentiality, we obtained protein sequences in FASTA format from NCBI RefSeq [16]for* B. subtilis, E. coli*, and *S. cerevisiae*. These organisms were chosen because their genomes have been sequenced and global knockout phenotype data was available [17-19]. We referred to these three species as 'query species,' since their respective proteins were the queries in the sequence alignments (Table 1). We referred to the proteins from the query species as 'query proteins.' Essentiality data was available for other organisms besides *B. subtilis*, *E. coli*, and *S. cerevisiae*, however, these data were not produced by complete gene deletion, as in *E. coli *and *S. cerevisiae*, or by insertion of a marker, as in *B. subtilis*, but by transposon mutagenesis (*Mycoplasma genitalium *[2,3], *Haemophilus influenzae *[20], *Escherichia coli (strain MG1655) *[21]) or anti-sense RNA (*Staphylococcus aureus (strains RN450 and RN4220) *[22]). Transposon mutagenesis can miss essential genes that tolerate transposon insertions as well as produce false negatives due to non-polar insertions. Inhibition by anti-sense RNA is a 'knock down' rather than a knockout of a gene and may not result in the complete removal of the transcript of the target gene. Also, this technique is limited to genes for which adequate expression of the anti-sense RNA can be obtained [17,18]. With these considerations in mind, we performed our analyses with *B. subtilis*, *E. coli*, and *S. cerevisiae *as the essentiality data for these three organisms were potentially more reliable.

**Table 1:** Selected query species. The three query species that had completed genome projects and complete global knockout data available

**Query Species**	**Domain**	**Taxonomy ID**	**Number of Proteins from NCBI RefSeq**	**Essential Genes that could be mapped to a NCBI RefSeq ID:**
*Bacillus subtilis (strain 168)*	Bacteria	224308	4105	271/271
*Escherichia coli (strain K12)*	Bacteria	83333	4237	299/303
*Saccharomyces cerevisiae*	Eukaryote	4932	5872	1050/1105

We also downloaded protein sequences, in FASTA format, for 22 bacterial and 15 eukaryote species with fully sequenced genomes. We referred to these species as 'subject species,' since their respective proteins were the subjects in the sequence alignments (Additional file 1). We referred to the proteins from the subject species as 'subject proteins.' All together, 14,214 query proteins (8342 bacterial and 5872 eukaryote) and 336,086 subject proteins (53,454 bacterial and 282,632 eukaryote) were analyzed.

**Figure 1:**
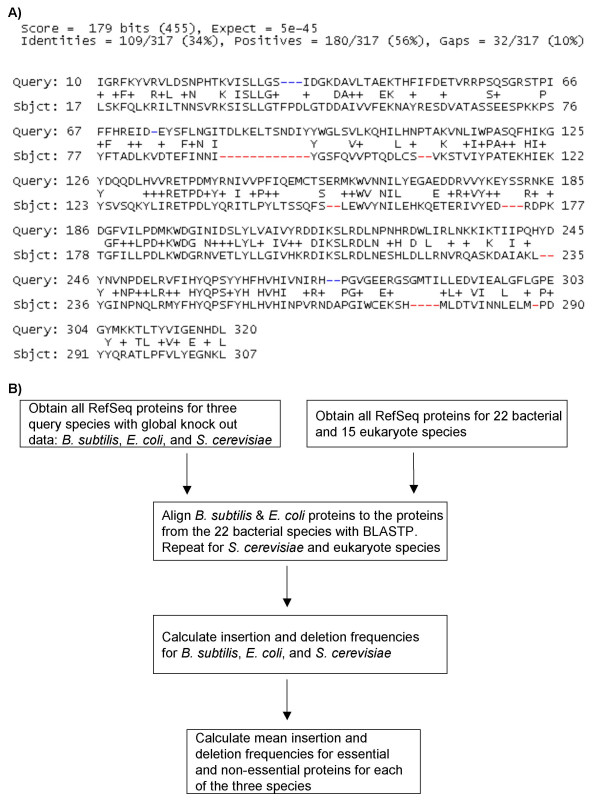
**Sample alignment and pipeline. A) Sample Alignment: **Gaps were reported as insertions/deletions with respect to the query sequence. There are seven insertions (red) and two deletions (blue) in this sample alignment. **B) Pipeline: **A summary of the steps taken to calculate the mean insertion and deletion frequencies for essential and non-essential proteins in *B. subtilis*, *E. coli*, and *S. cerevisiae*.

The comparison of the indel frequencies of essential and non-essential proteins was performed to determine if the frequencies differed in a statistically significant manner. We aligned all NCBI RefSeq proteins from *B. subtilis *and *E. coli *against the proteins of 22 bacteria subject species, and *S. cerevisiae *against the proteins from 15 eukaryote subject species with BLASTP. A gap opened in the query protein could be reported as a deletion in the query protein or as an insertion in the subject protein. Similarly, a gap opened in the subject protein could be reported as a deletion in the subject protein or as an insertion in the query protein. To maintain a consistent naming scheme, we reported gaps with respect to the query protein (Figure 1a). Figure 1b summarizes the steps performed while Additional file 2 shows a summary of the number of indels and proteins of high sequence similarity for each species-species comparison.

### Is there a significant difference between the indel frequencies of essential and non-essential proteins?

**Figure 2:**
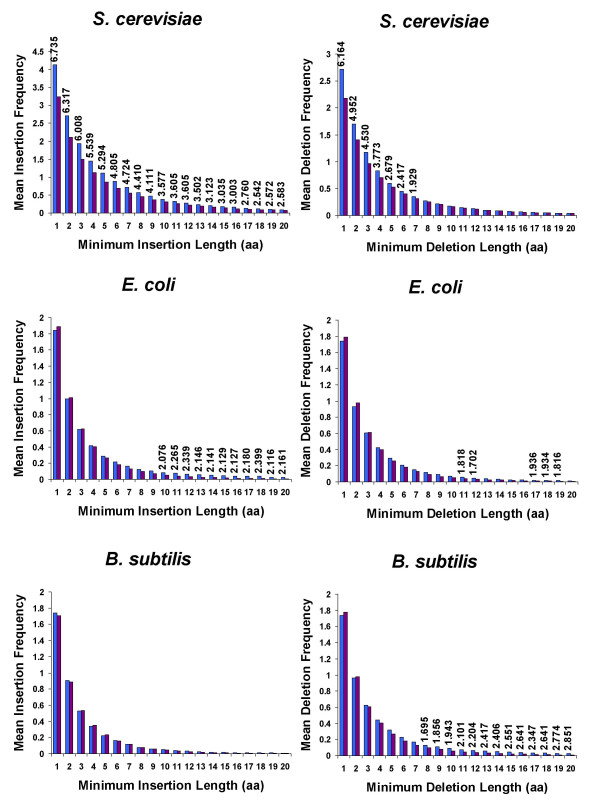
**Mean insertion and deletion frequencies in essential and non-essential proteins plotted against minimum indel length**. Mean insertion and deletion frequencies were calculated for essential and non-essential query proteins aligned to proteins from the 22 bacteria or 15 eukaryote species. The t-test statistic is shown for the minimum indel lengths that were found significantly more often in essential (blue bars) than non-essential (purple bars) proteins. Significance was set at P < 0.05. Note that no such difference was observed in insertions within *B. subtilis *proteins.

To evaluate whether or not the differences between mean indel frequencies of essential and non-essential proteins were statistically significant, we first calculated the frequencies of insertions and deletions of a given minimum length (one to twenty amino acids) for all query species (see Methods). Next, we calculated the mean insertion and deletion frequencies for both essential and non-essential proteins for each query species. Figure 2 contains plots of the mean insertion and deletion frequencies against the minimum insertion and deletion lengths for the three query species. As the figure illustrates, the mean frequencies in the proteins of the three query species decrease as the minimum indel lengths increase, suggesting that short indels are more likely to occur than long indels. Next, we performed t-tests to examine the null hypothesis that the mean indel frequencies of essential and non-essential proteins were equal. We observed that while the absolute differences between the mean indel frequencies were small, the differences were statistically significant as assessed by the t-test calculation (P < 0.05). As seen in the figure, the essential proteins in *B. subtilis*, *E. coli*, and *S. cerevisiae *had significantly different insertion and deletion frequencies from their non-essential counterparts. All significant t-test values were positive for the query species, which suggested that for these three organisms, essential proteins had a greater frequency of indels than non-essential proteins. While both insertions and deletions occurred significantly more often in essential proteins than in non-essential proteins for *E. coli *and *S. cerevisiae*, only deletions of minimum length eight to twenty amino acids produced significant results in *B. subtilis*.

It is interesting to note that while long indels in *S. cerevisiae *were significant, the greatest t-test value occurred when the indel length was defined as one or more amino acids. A large t-test value suggested that differences between the mean indel frequencies of essential and non-essential proteins were not likely due to chance alone. Furthermore, if indels of exactly one amino acid long were randomly distributed across essential and non-essential proteins, then one would expect that the t-test value of a longer minimum indel length would produce the greatest t-test value. However, this was not the case and one explanation for this trend could be that essential proteins contained a higher frequency of indels of exactly one amino acid in length. To test this possible explanation, we re-ran our BLASTP processing scripts again for *S. cerevisiae*, this time checking for indels of length exactly one to twenty amino acids. The results from this new set (data not shown) showed that there was significance even at the one amino acid indel length, and thus confirmed our suspicions.

While these initial t-test results supported our predictions that essential proteins of the three query species would have more indels than their respective non-essential proteins, we reasoned that the frequency of indels produced is at least partially dependent on the specific subject species chosen. To observe how our choice of subject species may have impacted our results, we repeated the t-test analysis with a smaller set of 14 randomly chosen subject species. After performing BLASTP of *B. subtilis *and *E. coli *against the proteins of nine sequenced bacterial species and *S. cerevisiae *against the proteins of five sequenced eukaryote species (Additional file 3), we observed similar trends in that essential proteins had significantly greater indel frequencies than non-essential proteins (P < 0.05) (Additional file 4). For example, in the complete set of bacterial subject proteins (22 bacteria species), *E. coli *insertions of minimum length 10 to 20 amino acids occurred more frequently in essential proteins than non-essential proteins, while with the smaller set of bacterial subject proteins, insertions of one and seven to twenty amino acids occurred more frequently in essential proteins. Similarly, in the complete set of bacterial subject proteins, *B. subtilis *deletions of minimum length eight to twenty amino acids occurred more frequently in essential proteins, while in the smaller set, this trend was extended to seven to twenty amino acids. These results showed that while the choice of subject species did alter the specific indel lengths that produced significant results, in general, the trends were consistent with our predictions. The only exception was the shorter insertions of *B. subtilis*. We observed that insertions of minimum length three, four, and six were found more frequently in non-essential proteins, as indicated by the negative t-test values. However, the longer deletions of *B. subtilis*, as discussed, followed the predicted trend. With this specific result in mind, we now speculate that perhaps only longer indels, say of length greater than or equaled to seven amino acids, are more likely to be found in essential proteins.

Another issue that may have impacted our initial t-test results was the quality of the protein sequences we used. A portion of the proteins we obtained from NCBI RefSeq resulted from computational predictions and/or have not undergone full manual curation. Therefore, sequencing and/or annotation errors of these protein sequences may have resulted in "pseudo-indels" in the BLASTP alignments. To observe how these proteins in the complete set of subject proteins from the 22 bacteria and 15 eukaryotes may have impacted our initial t-test results, we repeated the analyses and performed BLASTP of *S. cerevisiae *against the smaller set of five randomly chosen eukaryote subject species, but this time only fully curated and reviewed NCBI RefSeq proteins were included. We focused only on *S. cerevisiae *because all of its respective proteins in NCBI RefSeq were fully curated and reviewed, while this was not case for any of the proteins from the other two query species. If the resulting trends from this smaller set of subject species varied greatly with that which was observed with the complete set of subject species (15 eukaryotes), then it would be likely that the results produced from the complete set of subject proteins were caused by the pseudo-indels created by the alignments of the predicted and non-curated NCBI RefSeq proteins. However, this was not the case as the trends seen with the highly curated proteins were very similar to what was observed in the complete subject species set (Additional file 5). Therefore, we concluded that it was unlikely that the observed trend, in which the indel frequency of essential proteins was greater than that of non-essential proteins, was merely caused by sequencing and/or annotation errors. While we performed this check to further test our results, we wish to remind the reader that sequences in NCBI RefSeq represent a nearly non-redundant collection of sequences and is described as a 'summary' of the currently available information for each sequence [16].

### Cumulative insertion and deletion frequencies and approximation by the Weibull distribution

**Figure 3:**
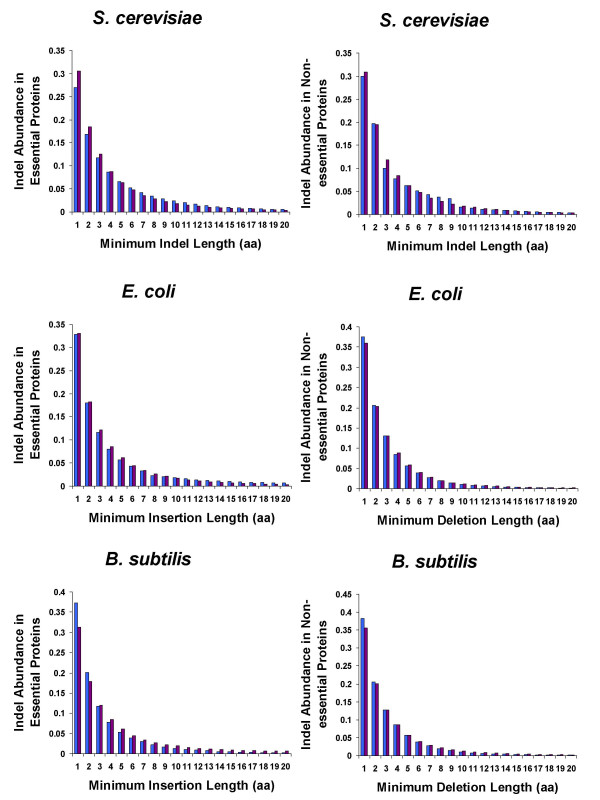
**Proportion of essential and non-essential proteins with indels plotted against minimum indel length**. Insertions are represented by blue bars while deletions are represented by purple bars.

To investigate if the abundance of indels in essential and non-essential proteins could be modeled by consistent statistical distributions, we calculated the cumulative distribution functions (*CDF*) for the minimum lengths of insertions and deletions in essential and non-essential proteins in the query species (Figure 3). As can be seen in Figure 3, the dependences between the abundance of indels in both essential and non-essential proteins and minimum indel lengths formed typical exponent-like distributions. In our previous work [10], we demonstrated that the distribution of indels of varying length across all proteins studied could be accurately described by the Weibull distribution:

*SDF(x) *= exp{-(x/α)^β^}, x ≥ 0, β > 0

where *SDF*(x) is the survival distribution function, α is a scaling factor, and β is a shape parameter that may reflect the evolutionary rates for the occurrence/expansion of indels in the proteomes examined. The Weibull distribution is a statistical function defined within extreme value theory and often used in reliability engineering to model material strength and durability of electronic and mechanical components [23]. The Weibull distribution utilizes a time-to-failure measure to assess the reliability of a system and to predict its stability. A typical time-to-failure experiment involves applying a disruptive stress to a sample of objects representative of the population. The time taken for each object to break (i.e. to fail) is recorded. The resulting values are then used to determine if the objects in the population follow a Weibull distribution. For example, a recent study [24] characterized the strength of three ceramic materials by applying mechanical stresses of 70 – 400 MPa/s to determine characteristics of breaking. Similarly, the formation and expansion of indels in the proteome of an organism take place under 'disruptive stress' (evolutionary pressure). An indel 'breaks' or 'fails' when it is lost. Because our previous Weibull analyses only considered indels across all proteins regardless of their essentiality [10], we examined whether the statistical function could accurately describe the abundance of indels in essential and non-essential proteins separately. For each query species the double logarithmic transformation of *SDF*(x), as represented by the *CDF*, was calculated and plotted:

log(-log(*SDF(x)*) = βlog(x) - βlog(α)

**Figure 4:**
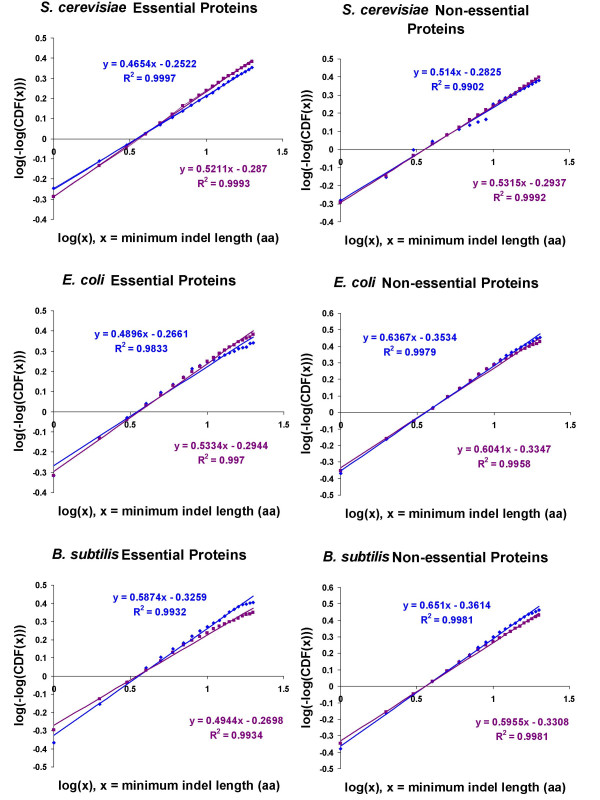
Approximation of abundance of indels with the Weibull distribution. r^2 ^values close to 1.0 indicated that the abundance of insertions (blue points and blue line) and deletions (purple points and purple line) in essential and non-essential proteins of the three query species could be accurately modeled by the Weibull distribution.

If the abundance of indels in the three query species could be accurately described by this distribution, then the resulting plots should be linear. We observed that the Weibull distribution could accurately model the dependence between the length of indels and their abundance in the essential and non-essential proteins in the query species, as indicated by the high r^2 ^values (Figure 4). The β parameter is represented by the slopes in each of the graphs in Figure 4 and the values suggested two observations. Firstly, as described previously [25], a β value of less than one indicates that there is reliable growth in the system as the rate of failure is decreasing. In this case, our results indicated that some indels are retained over evolutionary time, suggesting some functional importance. Secondly, while the differences between the β values of essential and non-essential proteins are small, the non-essential proteins in all three query species have greater β values for both insertions and deletions, suggesting that indels occur and expand more readily in non-essential proteins. This observation appeared to be at odds with our earlier observations on the mean indel frequency of essential and non-essential proteins. We wondered how it could be possible for non-essential proteins to acquire and expand their indels at a slightly faster rate and yet, in general, observe more indels in essential proteins. This observation may be explained by the differences in the evolutionary age of essential and non-essential genes. A recent study into two fungal species [26], *Schizosaccharomyces pombe *and *Saccharomyces cerevisiae*, showed that the more ancient a gene was, the more likely it was to be essential. Thus, essential genes may have had more time to accumulate and expand their indels.

### Can indel frequencies be used to discriminate between an essential and non-essential protein?

While the t-test statistic assesses whether or not the difference in the means of a quantifiable trait from two populations is significant, it does not take into consideration the actual magnitude of the difference. Even if the mean indel frequency of essential proteins was statistically different from that of non-essential proteins, if there was a large amount of overlap between the two distributions, it would still be difficult to predict whether a protein was essential or not based merely on its indel frequency. To determine if indel frequencies could be used as a marker to differentiate between essential and non-essential proteins, Receiver Operating Characteristic (ROC) curves were utilized. The Area Under the ROC curve (AUROC) was used as an assessment of the accuracy of the predictions. An AUROC of 1.0 implies that all predictions were correct, suggesting that all essential proteins can be completely separated from non-essential proteins based on some indel frequency threshold. An AUROC of 0.50 suggests that using indel frequency to predict essentiality has 50% sensitivity and specificity, which is not a useful test. Finally, an AUROC that is less than 0.50 implies that the opposite trend, in which non-essential proteins have a higher frequency of indels than essential proteins, is observed.

We calculated AUROCs for each of the query species. Similar to the t-tests, each of the query species was compared to the other species in the same domain. The AUROC results for all three query species were moderate as *S. cerevisiae *was the only query species to produce AUROCs between 0.57 to 0.59, while *B. subtilis *and *E. coli *AUROC values ranged from 0.46 to 0.56 (data not shown). These weak trends were not unexpected, because our reasoning also allowed non-essential proteins to use indels as a way to acquire and/or modify protein-protein interactions.

While our t-tests showed that essential proteins have significantly more indels than non-essential proteins, these AUROC results showed that indels were found frequently enough in non-essential proteins to make it difficult to accurately predict whether a protein is essential or not based solely on its indel frequency. A recent publication [27] identified 14 characteristic sequence features, such as codon adaptation, hydrophobicity, and localization signals, which are potentially associated with essential genes in fungal genomes. Thus, many different features are likely indicative of essential proteins and perhaps the predictions based on indel frequency would be more accurate if these other features were considered.

### Do proteins with indels have different network properties than those without indels?

It has been well documented that essential proteins are often involved in a greater number of interactions (i.e. a greater connectivity) than non-essential proteins [28,29]. Because indels tend to occur on the external surface of proteins, usually as reverse turns or coils within loops [30,31], and these structures play important roles in protein-protein interactions, we reasoned that formation of indels could be a means by which proteins acquired and/or modified the interactions that they are involved in. Using the protein-protein interaction counts for the 4148 *S. cerevisiae *proteins available from the Munich Information Center for Protein Sequences (MIPS) database [32], we determined whether indel containing proteins in *S. cerevisiae *had a greater mean connectivity than those that do not. We calculated the mean connectivity of proteins with and without indels of minimum length of four and ten amino acids (Table 2). While the absolute differences between the mean connectivity of both types of proteins were small, the differences were statistically significant (P < 0.05) as determined by the t-test. Therefore, in general, proteins with indels have more connections than proteins that do not. This can be explained by indels creating and/or exposing new interaction sites, which result in new interactions, as was illustrated in the *L. donovani *example [7].

**Table 2:** Summary of mean connectivity and betweenness of *S. cerevisiae *proteins with and without indels: The mean connectivity and betweenness of indel containing proteins were significantly greater than those of the non-indel containing proteins. Significance was set at P < 0.05

Min Indel Length (aa)	Number of proteins with at least one indel of at least 4 or 10 aa long	Mean connectivity of proteins with at least one indel of at least 4 or 10 aa long	Number of proteins without at least one indel of at least 4 or 10 aa long	Mean connectivity of proteins without at least one indel of at least 4 or 10 aa long	Betweenness of proteins with at least one indel of at least 4 or 10 aa long	Betweenness of proteins without at least one indel of at least 4 or 10 aa long
4	907	4.194	562	3.986	15354	15133
10	381	4.394	1088	4.017	15712	15115

We also considered whether indel containing proteins had a greater betweenness than proteins without indels. The betweenness is a measure in graph theory and is determined by counting the number of times a particular vertex is located on the shortest path between two vertices in a network [33]. From a biological perspective, the betweenness accounts for the direct and indirect influences of proteins at a distant location in the network. For example, if two clusters of interacting proteins, A and B, are joined together only through their mutual interaction with protein X, then X would have a high betweenness measure, because if any protein in A is to interact with another protein in B, it must do so through a direct or indirect interaction with protein X.

The naïve method used to calculate the betweenness measure can require up to O(n^3^) in time and O(n^2^) in space, making the calculation inefficient. Therefore, we used a faster method developed by Brandes [33], which we implemented in C and executed under a Linux platform. Briefly, this method calculates the betweenness for a particular vertex, v, by first computing the number of times v occurs between any other two vertices, x and y, in the network. Next, a value known as the pair-dependency is calculated. This value is the proportion of shortest paths between vertices x and y that v lines on. This step is repeated for all vertices v, x, and y and the values are summed. Table 2 shows that indel containing proteins had greater betweenness, suggesting their importance in the *S. cerevisiae *protein-protein interaction network. Taken together, these two observations suggested that the presence of indels is related to two network properties (connectivity and betweenness) of proteins in PINs. One application of these results would be in bait-prey pull down experiments. These results suggest that to increase the coverage of the PIN with each pull down experiment, the bait should be one that contains an indel, as indel containing proteins are involved in a greater number of interactions and have greater betweenness.

## Conclusion

We previously conducted a large scale analysis of potentially targetable indels in bacterial and protozoan pathogen proteins [10]. In that study, we located many examples of essential pathogen proteins that contained sizable indels. Therefore, the objective of this study was to determine how indels were related to essential and non-essential proteins. To our knowledge, such a relationship had not been previously explored. We further analyzed indels for their ability to discriminate between essential and non-essential proteins and compared two network properties, connectivity and betweenness, of indel and non-indel containing proteins. We determined that for three species, *Bacillus subtilis, Escherichia coli*, and *Saccharomyces cerevisiae*, essential proteins had a greater mean indel frequency than non-essential proteins. The abundance of indels in both types of proteins could be accurately modeled by the Weibull distribution. Furthermore, we demonstrated with ROC curves that accurate discrimination of essential and non-essential proteins based solely on indel frequency could not be achieved. Finally, we showed that indel containing proteins had different network properties, namely that they had greater connectivity and betweenness, suggesting a possible role of indels in the regulation of interaction partners.

In our analyses, we did not consider the actual location of the indels in the folded three dimensional protein structures, which is critical for effective drug design. Therefore, some future directions that we will focus on include three dimensional modeling of indel containing proteins as well identifying any functional protein domains that are commonly disrupted by indels. Given that indels can be used to selectively target essential pathogen proteins that have high sequence similarity to human proteins, characterization of these indels will potentially lead to new drug targets for infectious diseases.

## Methods

### Systematic knockout data and NCBI RefSeq proteins

We conducted a broad literature search to identify fully sequenced genomes in which genome-wide knockout data was available (i.e. protein essentiality is defined). We located complete knockout data for *B. subtilis (strain 168) *[17], *E. coli (strain K12) *[18], and *S. cerevisiae *[19]. For each of these species, we downloaded the complete non-redundant set of proteins ('query proteins') in FASTA format from NCBI RefSeq [16]. In total, 14,214 query proteins were analyzed. Next, we obtained the list of essential genes and cross referenced them to a NCBI RefSeq protein ID using an in-house Perl script that utilized BioPerl modules (Version 1.5.1) [34] to search for the gene name in the complete set of RefSeq Genbank protein files for the particular query organism.

### NCBI RefSeq proteins for BLAST databases

We searched the Entrez Genome Project section of NCBI [35] for all bacterial and eukaryote genome projects annotated as completed. From this list, a wide range of bacterial and eukaryote species were chosen. We chose species from a wide range of different classes to avoid biasing our results to particular organisms in a specific class. This resulted in 22 bacterial species and 15 eukaryote species (Additional file 1). Next, we obtained the complete set of protein sequences from these selected organisms ('subject proteins') from NCBI RefSeq. In total, this set consisted of 53,454 bacterial and 282,632 eukaryote subject proteins.

To further validate our results, we randomly chose nine bacterial and five eukaryote species (Additional file 3) and obtained their respective proteins from NCBI RefSeq (35,429 bacterial and 75,881 eukaryote). We also obtained the fully curated and reviewed proteins for each of the five eukaryote species (54,927 reviewed eukaryote proteins).

### BLASTP parameters used to determine alignments

We used formatdb [36] to format the subject protein sequences into BLAST databases. To align the *B. subtilis, E. coli*, and *S. cerevisiae *query proteins to the subject proteins, we conducted BLASTP-based alignment of *B. subtilis *and *E. coli *query proteins against the 53,454 bacterial subject proteins and *S. cerevisiae *query proteins against the 282,632 eukaryote subject proteins. We set a maximum E-value of 10^-5 ^and considered only sequence alignments with a minimum 50% similarity. The same parameters were used for the analyses with the smaller set of subject species. The BLASTP alignments were performed on nine IBM machines running the CentOS Linux operating system.

### Processing alignments that contain indels

We developed in-house Perl scripts that would process the results of the BLASTP alignments and search for indels. For all alignments that matched our BLASTP parameters, we searched for gaps that were opened in the query protein (deletions) and the subject protein (insertions) of minimum X amino acids long, where the values of X ranged from one to twenty amino acids. Note that gaps were reported as insertions or deletions based on the query protein (Figure 1a). For each insertion of minimum X amino acids long, we calculated the Insertion Frequency (*IF*) as follows:

IF=I1+I2+I3+...+I22H1+H2+H3+...+H22
 MathType@MTEF@5@5@+=feaafiart1ev1aaatCvAUfKttLearuWrP9MDH5MBPbIqV92AaeXatLxBI9gBaebbnrfifHhDYfgasaacH8akY=wiFfYdH8Gipec8Eeeu0xXdbba9frFj0=OqFfea0dXdd9vqai=hGuQ8kuc9pgc9s8qqaq=dirpe0xb9q8qiLsFr0=vr0=vr0dc8meaabaqaciaacaGaaeqabaqabeGadaaakeaacqWGjbqscqWGgbGrcqGH9aqpdaWcaaqaaiabdMeajnaaBaaaleaacqaIXaqmaeqaaOGaey4kaSIaemysaK0aaSbaaSqaaiabikdaYaqabaGccqGHRaWkcqWGjbqsdaWgaaWcbaGaeG4mamdabeaakiabgUcaRiabc6caUiabc6caUiabc6caUiabgUcaRiabdMeajnaaBaaaleaacqaIYaGmcqaIYaGmaeqaaaGcbaGaemisaG0aaSbaaSqaaiabigdaXaqabaGccqGHRaWkcqWGibasdaWgaaWcbaGaeGOmaidabeaakiabgUcaRiabdIeainaaBaaaleaacqaIZaWmaeqaaOGaey4kaSIaeiOla4IaeiOla4IaeiOla4Iaey4kaSIaemisaG0aaSbaaSqaaiabikdaYiabikdaYaqabaaaaaaa@5044@

where *I*_*i *_is the number of insertions the query species shares with species *i *and *H*_*i *_is the number of proteins that satisfied our alignment parameters between the query species and species *i*. Similarly, we calculated the Deletion Frequency (*DF*) as follows:

DF=D1+D2+D3+...+D22H1+H2+H3+...+H22
 MathType@MTEF@5@5@+=feaafiart1ev1aaatCvAUfKttLearuWrP9MDH5MBPbIqV92AaeXatLxBI9gBaebbnrfifHhDYfgasaacH8akY=wiFfYdH8Gipec8Eeeu0xXdbba9frFj0=OqFfea0dXdd9vqai=hGuQ8kuc9pgc9s8qqaq=dirpe0xb9q8qiLsFr0=vr0=vr0dc8meaabaqaciaacaGaaeqabaqabeGadaaakeaacqWGebarcqWGgbGrcqGH9aqpdaWcaaqaaiabdseaenaaBaaaleaacqaIXaqmaeqaaOGaey4kaSIaemiraq0aaSbaaSqaaiabikdaYaqabaGccqGHRaWkcqWGebardaWgaaWcbaGaeG4mamdabeaakiabgUcaRiabc6caUiabc6caUiabc6caUiabgUcaRiabdseaenaaBaaaleaacqaIYaGmcqaIYaGmaeqaaaGcbaGaemisaG0aaSbaaSqaaiabigdaXaqabaGccqGHRaWkcqWGibasdaWgaaWcbaGaeGOmaidabeaakiabgUcaRiabdIeainaaBaaaleaacqaIZaWmaeqaaOGaey4kaSIaeiOla4IaeiOla4IaeiOla4Iaey4kaSIaemisaG0aaSbaaSqaaiabikdaYiabikdaYaqabaaaaaaa@5012@

where *D*_*i *_is the number of deletions the query species shared with species *i *and *H*_*i *_is the number of proteins that satisfied our alignment parameters between the query species and species *i*. Note that for *S. cerevisiae *as the query species, *I*_22_, *H*_22_, and *D*_22 _would be *I*_15_, *H*_15_, and *D*_15_, respectively, as there was only 15 eukaryote subject species.

### Calculations and statistical analyses

Receiver Operator Characteristic (ROC) curves and the corresponding Area Under the ROC curve (AUROC) were determined using the R statistical package, version 2.3.1 [37] for Linux-like operating systems and the ROCR package [38]. An ROC curve plots the Sensitivity (True Positives/(True Positives + False Negatives)) vs False Positive Rate (1 - (True Negatives)/(True Negatives + False Positives)). Perl scripts performing t-test calculations were also implemented and significance was set at P < 0.05.

### Protein-protein interaction counts

The *S. cerevisiae *protein-protein interaction counts were obtained from the Munich Information Center for Protein Sequences (MIPS) database [32]. In total, we obtained interaction counts for 4148 proteins. Of the 4148 *S. cerevisiae *proteins with interaction counts, 837 (20.2%) were essential. We determined the best match in *Homo sapiens *using the BLASTP algorithm. Again, we specified a maximum E-value of 10^-5 ^and that the query and subject proteins shared at least 50% sequence similarity. Using in-house Perl scripts, we then determined which proteins contained at least one indel of at least four and ten amino acids long.

## Authors' contributions

SKC acquired the data from various online resources, developed the computer code, performed the analyses, and wrote the manuscript. FH implemented the betweenness algorithm and performed the network analyses. AC conceived of the study, while SKC, MH, and AC participated in its design and interpretation of results. All authors read and approved the final manuscript.

## Supplementary Material

Additional File 1 The 22 bacterial and 15 eukaryote subject species utilized. Click here for file

Additional File 2 Indel and similar protein counts for each query species when compared to each subject species. Click here for file

Additional File 3 The nine bacteria and five eukaryote subject species utilized. Click here for file

Additional File 4 **Mean insertion and deletion frequencies in essential and non-essential proteins plotted against minimum indel length**. Mean insertion and deletion frequencies were calculated for essential and non-essential query proteins aligned to the proteins of the 14 randomly chosen subject species. The t-test statistic is shown for the minimum indel lengths that were found significantly more often in essential (blue bars) than non-essential (purple bars) proteins. Significance was set at P < 0.05. Note that no such difference was observed in deletions within *E. coli *proteins. Also note that insertions of minimum length three, four, and six amino acids were found more frequently in non-essential than essential proteins of *B. subtilis*. See text for discussion. Click here for file

Additional File 5 **Mean indel frequency calculated with curated eukaryote proteins. **Mean indel frequencies were calculated for the curated *S. cerevisiae *essential and non-essential proteins aligned to the curated proteins of the five randomly chosen subject species. Note that the observed trend in which the mean indel frequency of essential proteins was greater than that of non-essential proteins was also seen with this smaller set of curated proteins, suggesting that the observed trend seen with the proteins from the complete set of subject species was not merely due to sequencing/annotation errors. The t-test statistic is shown for the minimum indel lengths that were found significantly more often in essential (blue bars) than non-essential (purple bars) proteins. Significance was set at P < 0.05. See text for discussion. Click here for file
