# Supplementary material for: Relationship between insertion/deletion (indel) frequency of proteins and essentiality
Source: BMC Bioinformatics. 2007 Jun 28;8:227. doi: 10.1186/1471-2105-8-227 (PMC1925122; doi:10.1186/1471-2105-8-227)
Supplement: Additional File 1 — The 22 bacterial and 15 eukaryote subject species utilized. [file 1471-2105-8-227-S1.ppt]

## Slide 1
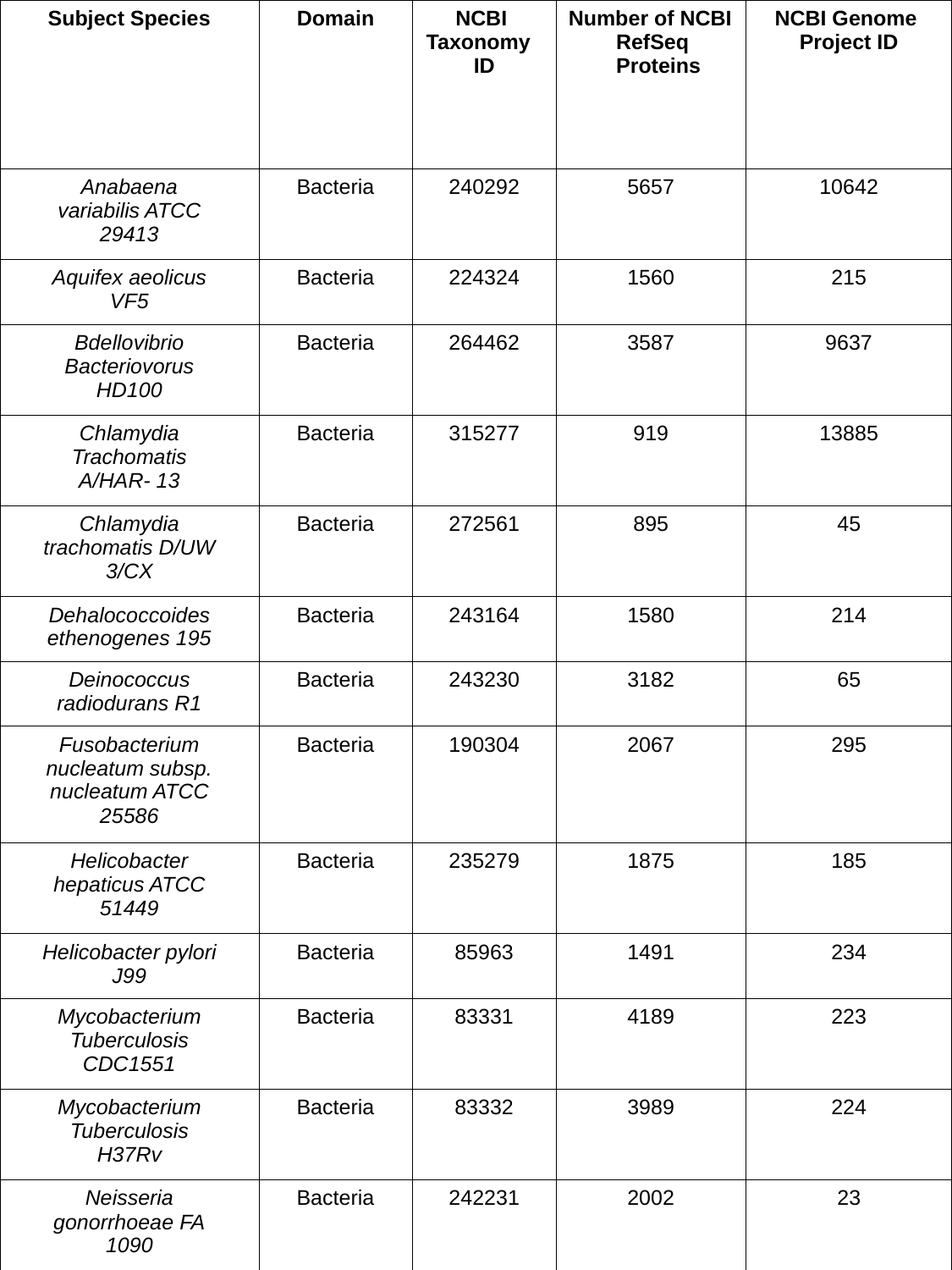

| Subject Species | Domain | NCBI Taxonomy ID | Number of NCBI RefSeq Proteins | NCBI Genome Project ID |
| --- | --- | --- | --- | --- |
| Anabaena variabilis ATCC 29413 | Bacteria | 240292 | 5657 | 10642 |
| Aquifex aeolicus VF5 | Bacteria | 224324 | 1560 | 215 |
| Bdellovibrio Bacteriovorus HD100 | Bacteria | 264462 | 3587 | 9637 |
| Chlamydia Trachomatis A/HAR- 13 | Bacteria | 315277 | 919 | 13885 |
| Chlamydia trachomatis D/UW 3/CX | Bacteria | 272561 | 895 | 45 |
| Dehalococcoides ethenogenes 195 | Bacteria | 243164 | 1580 | 214 |
| Deinococcus radiodurans R1 | Bacteria | 243230 | 3182 | 65 |
| Fusobacterium nucleatum subsp. nucleatum ATCC 25586 | Bacteria | 190304 | 2067 | 295 |
| Helicobacter hepaticus ATCC 51449 | Bacteria | 235279 | 1875 | 185 |
| Helicobacter pylori J99 | Bacteria | 85963 | 1491 | 234 |
| Mycobacterium Tuberculosis CDC1551 | Bacteria | 83331 | 4189 | 223 |
| Mycobacterium Tuberculosis H37Rv | Bacteria | 83332 | 3989 | 224 |
| Neisseria gonorrhoeae FA 1090 | Bacteria | 242231 | 2002 | 23 |
| Neisseria meningitidis MC58 | Bacteria | 122586 | 2063 | 251 |
| Neisseria meningitidis Z2491 | Bacteria | 122587 | 2065 | 252 |
| Rickettsia prowazekii str. Madrid E | Bacteria | 272947 | 835 | 43 |
| Staphylococcus aureus COL | Bacteria | 93062 | 2618 | 238 |
| Staphylococcus aureus subsp. aureus Mu50 | Bacteria | 158878 | 2731 | 263 |
| Staphylococcus aureus subsp. aureus MW2 | Bacteria | 196620 | 2632 | 306 |
| Staphylococcus aureus subsp. aureus NCTC 8325 | Bacteria | 93061 | 2892 | 237 |
| Thermotoga maritima MSB8 | Bacteria | 243274 | 1858 | 111 |
| Treponema denticola ATCC 35405 | Bacteria | 243275 | 2767 | 4 |
| Arabidopsis thaliana | Eukaryote | 3702 | 30480 | 9506 |
| Caenorhabditis elegans | Eukaryote | 6239 | 22844 | 9548 |
| Candida glabrata CBS 138 | Eukaryote | 284593 | 5181 | 12362 |
| Cryptococcus neoformans var. neoformans JEC21 | Eukaryote | 214684 | 6475 | 9581 |
| Debaryomyces hansenii CBS767 | Eukaryote | 284592 | 6317 | 12365 |
| Drosophila melanogaster | Eukaryote | 7227 | 19816 | 9554 |
| Entamoeba histolytica HM-1:IMSS | Eukaryote | 294381 | 9772 | 9532 |
| Eremothecium gossypii | Eukaryote | 33169 | 9436 | 10630 |
| Homo sapiens | Eukaryote | 9606 | 40877 | 9558 |
| Kluyveromyces lactis NRRL Y-1140 | Eukaryote | 284590 | 5326 | 12363 |
| Mus musculus | Eukaryote | 10090 | 58794 | 9559 |
| Oryza sativa | Eukaryote | 4530 | 36142 | 9512 |
| Schizosaccharomyces pombe | Eukaryote | 284812 | 5045 | 9517 |
| Trypanosoma cruzi | Eukaryote | 5693 | 19607 | 9530 |
| Yarrowia lipolytica CLIB99 | Eukaryote | 284591 | 6520 | 12364 |
