# Supplementary material for: Relationship between insertion/deletion (indel) frequency of proteins and essentiality
Source: BMC Bioinformatics. 2007 Jun 28;8:227. doi: 10.1186/1471-2105-8-227 (PMC1925122; doi:10.1186/1471-2105-8-227)
Supplement: Additional File 2 — Indel and similar protein counts for each query species when compared to each subject species. [file 1471-2105-8-227-S2.ppt]

## Slide 1
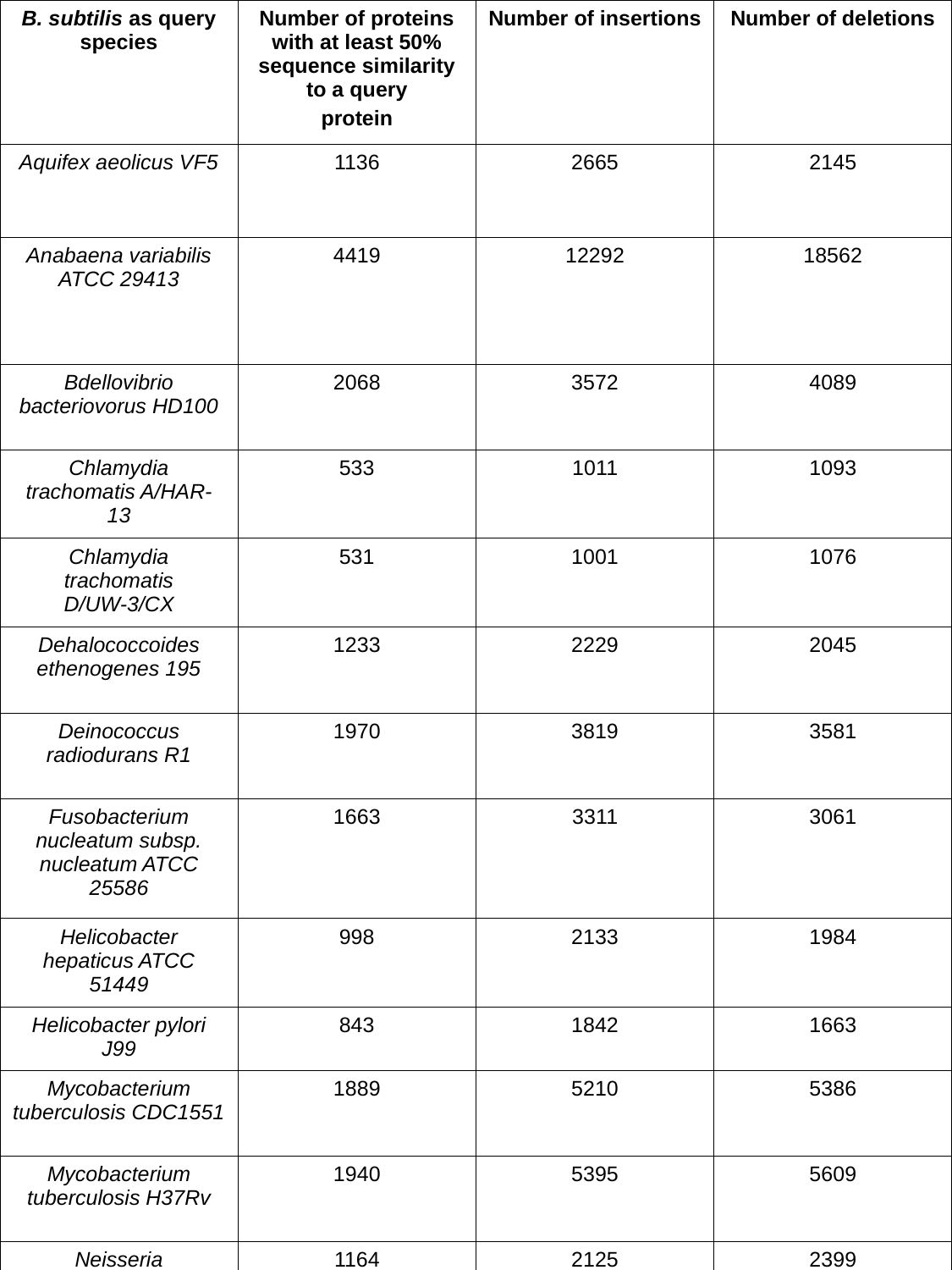

| B. subtilis as query species | Number of proteins with at least 50% sequence similarity to a query protein | Number of insertions | Number of deletions |
| --- | --- | --- | --- |
| Aquifex aeolicus VF5 | 1136 | 2665 | 2145 |
| Anabaena variabilis ATCC 29413 | 4419 | 12292 | 18562 |
| Bdellovibrio bacteriovorus HD100 | 2068 | 3572 | 4089 |
| Chlamydia trachomatis A/HAR- 13 | 533 | 1011 | 1093 |
| Chlamydia trachomatis D/UW-3/CX | 531 | 1001 | 1076 |
| Dehalococcoides ethenogenes 195 | 1233 | 2229 | 2045 |
| Deinococcus radiodurans R1 | 1970 | 3819 | 3581 |
| Fusobacterium nucleatum subsp. nucleatum ATCC 25586 | 1663 | 3311 | 3061 |
| Helicobacter hepaticus ATCC 51449 | 998 | 2133 | 1984 |
| Helicobacter pylori J99 | 843 | 1842 | 1663 |
| Mycobacterium tuberculosis CDC1551 | 1889 | 5210 | 5386 |
| Mycobacterium tuberculosis H37Rv | 1940 | 5395 | 5609 |
| Neisseria gonorrhoeae FA 1090 | 1164 | 2125 | 2399 |
| Neisseria meningitidis MC58 | 1209 | 2150 | 2499 |
| Neisseria meningitidis Z2491 | 1197 | 2128 | 2502 |
| Rickettsia prowazekii str. Madrid E | 602 | 1153 | 1316 |
| Staphylococcus aureus COL | 2949 | 5201 | 4689 |
| Staphylococcus aureus subsp. aureus Mu50 | 3015 | 5333 | 4850 |
| Staphylococcus aureus subsp. aureus MW2 | 2961 | 5215 | 4709 |
| Staphylococcus aureus subsp. aureus NCTC 8325 | 2978 | 5216 | 4728 |
| Thermotoga maritima MSB8 | 1909 | 4395 | 3395 |
| Treponema denticola ATCC 35405 | 1534 | 2756 | 3090 |

## Slide 2
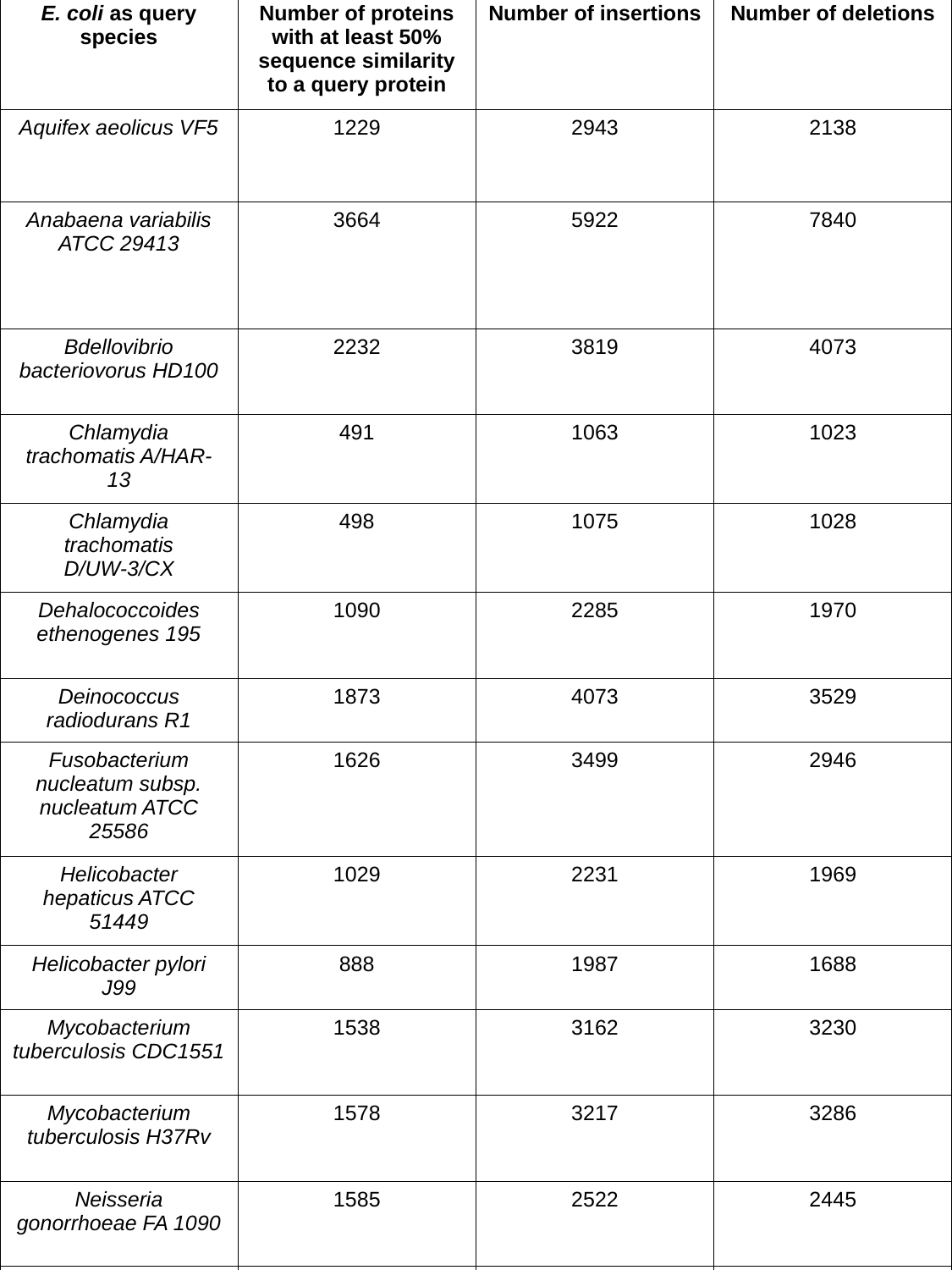

| E. coli as query species | Number of proteins with at least 50% sequence similarity to a query protein | Number of insertions | Number of deletions |
| --- | --- | --- | --- |
| Aquifex aeolicus VF5 | 1229 | 2943 | 2138 |
| Anabaena variabilis ATCC 29413 | 3664 | 5922 | 7840 |
| Bdellovibrio bacteriovorus HD100 | 2232 | 3819 | 4073 |
| Chlamydia trachomatis A/HAR- 13 | 491 | 1063 | 1023 |
| Chlamydia trachomatis D/UW-3/CX | 498 | 1075 | 1028 |
| Dehalococcoides ethenogenes 195 | 1090 | 2285 | 1970 |
| Deinococcus radiodurans R1 | 1873 | 4073 | 3529 |
| Fusobacterium nucleatum subsp. nucleatum ATCC 25586 | 1626 | 3499 | 2946 |
| Helicobacter hepaticus ATCC 51449 | 1029 | 2231 | 1969 |
| Helicobacter pylori J99 | 888 | 1987 | 1688 |
| Mycobacterium tuberculosis CDC1551 | 1538 | 3162 | 3230 |
| Mycobacterium tuberculosis H37Rv | 1578 | 3217 | 3286 |
| Neisseria gonorrhoeae FA 1090 | 1585 | 2522 | 2445 |
| Neisseria meningitidis MC58 | 1590 | 2468 | 2476 |
| Neisseria meningitidis Z2491 | 1576 | 2399 | 2401 |
| Rickettsia prowazekii str. Madrid E | 628 | 1224 | 1062 |
| Staphylococcus aureus COL | 1998 | 3963 | 3739 |
| Staphylococcus aureus subsp. aureus Mu50 | 2057 | 4046 | 3803 |
| Staphylococcus aureus subsp. aureus MW2 | 2035 | 4053 | 3810 |
| Staphylococcus aureus subsp. aureus NCTC 8325 | 2044 | 4016 | 3767 |
| Thermotoga maritima MSB8 | 1827 | 4349 | 3713 |
| Treponema denticola ATCC 35405 | 1376 | 2741 | 2950 |

## Slide 3
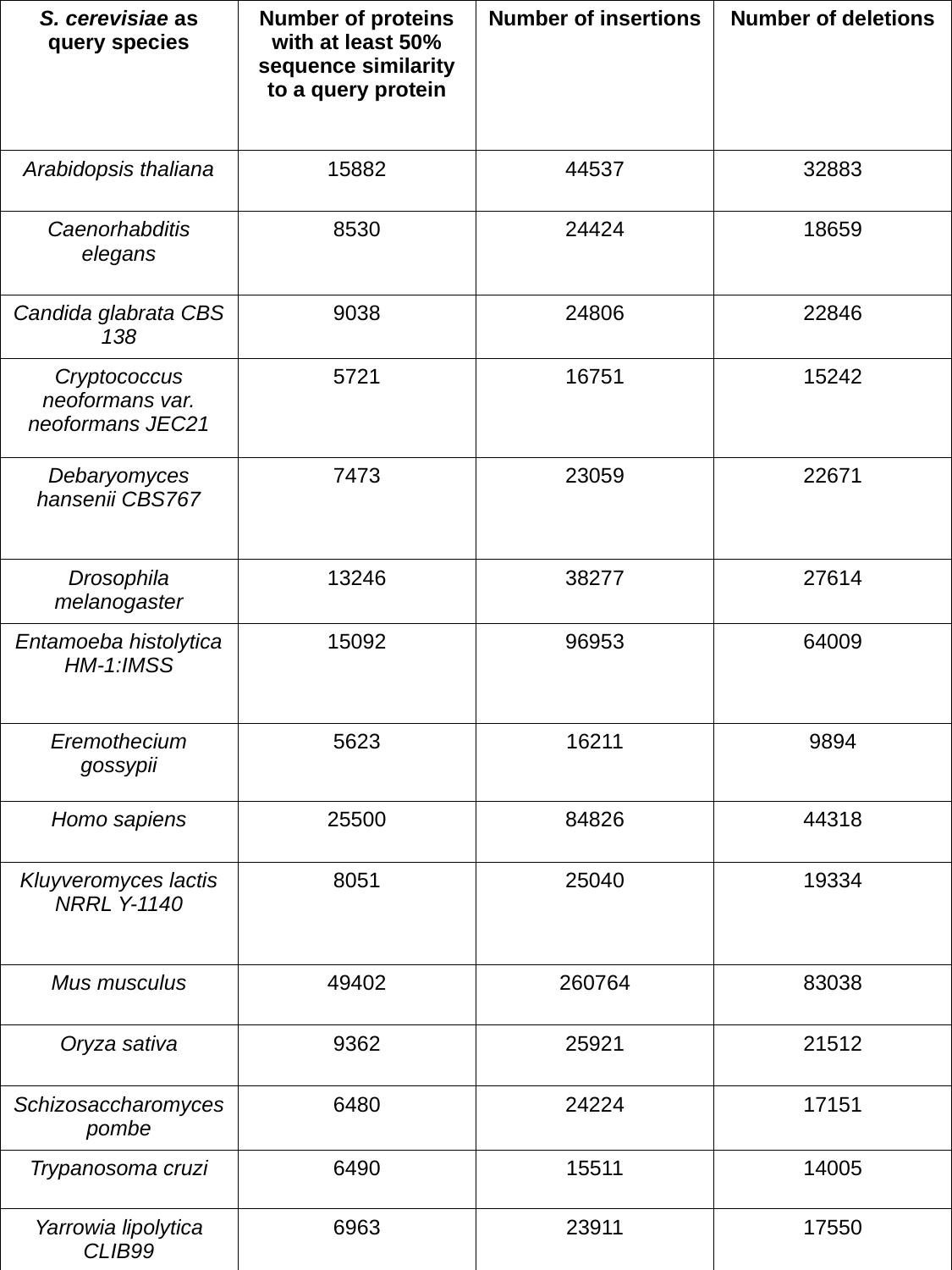

| S. cerevisiae as query species | Number of proteins with at least 50% sequence similarity to a query protein | Number of insertions | Number of deletions |
| --- | --- | --- | --- |
| Arabidopsis thaliana | 15882 | 44537 | 32883 |
| Caenorhabditis elegans | 8530 | 24424 | 18659 |
| Candida glabrata CBS 138 | 9038 | 24806 | 22846 |
| Cryptococcus neoformans var. neoformans JEC21 | 5721 | 16751 | 15242 |
| Debaryomyces hansenii CBS767 | 7473 | 23059 | 22671 |
| Drosophila melanogaster | 13246 | 38277 | 27614 |
| Entamoeba histolytica HM-1:IMSS | 15092 | 96953 | 64009 |
| Eremothecium gossypii | 5623 | 16211 | 9894 |
| Homo sapiens | 25500 | 84826 | 44318 |
| Kluyveromyces lactis NRRL Y-1140 | 8051 | 25040 | 19334 |
| Mus musculus | 49402 | 260764 | 83038 |
| Oryza sativa | 9362 | 25921 | 21512 |
| Schizosaccharomyces pombe | 6480 | 24224 | 17151 |
| Trypanosoma cruzi | 6490 | 15511 | 14005 |
| Yarrowia lipolytica CLIB99 | 6963 | 23911 | 17550 |
