# Supplementary material for: Relationship between insertion/deletion (indel) frequency of proteins and essentiality
Source: BMC Bioinformatics. 2007 Jun 28;8:227. doi: 10.1186/1471-2105-8-227 (PMC1925122; doi:10.1186/1471-2105-8-227)
Supplement: Additional File 3 — The nine bacteria and five eukaryote subject species utilized. [file 1471-2105-8-227-S3.ppt]

## Slide 1
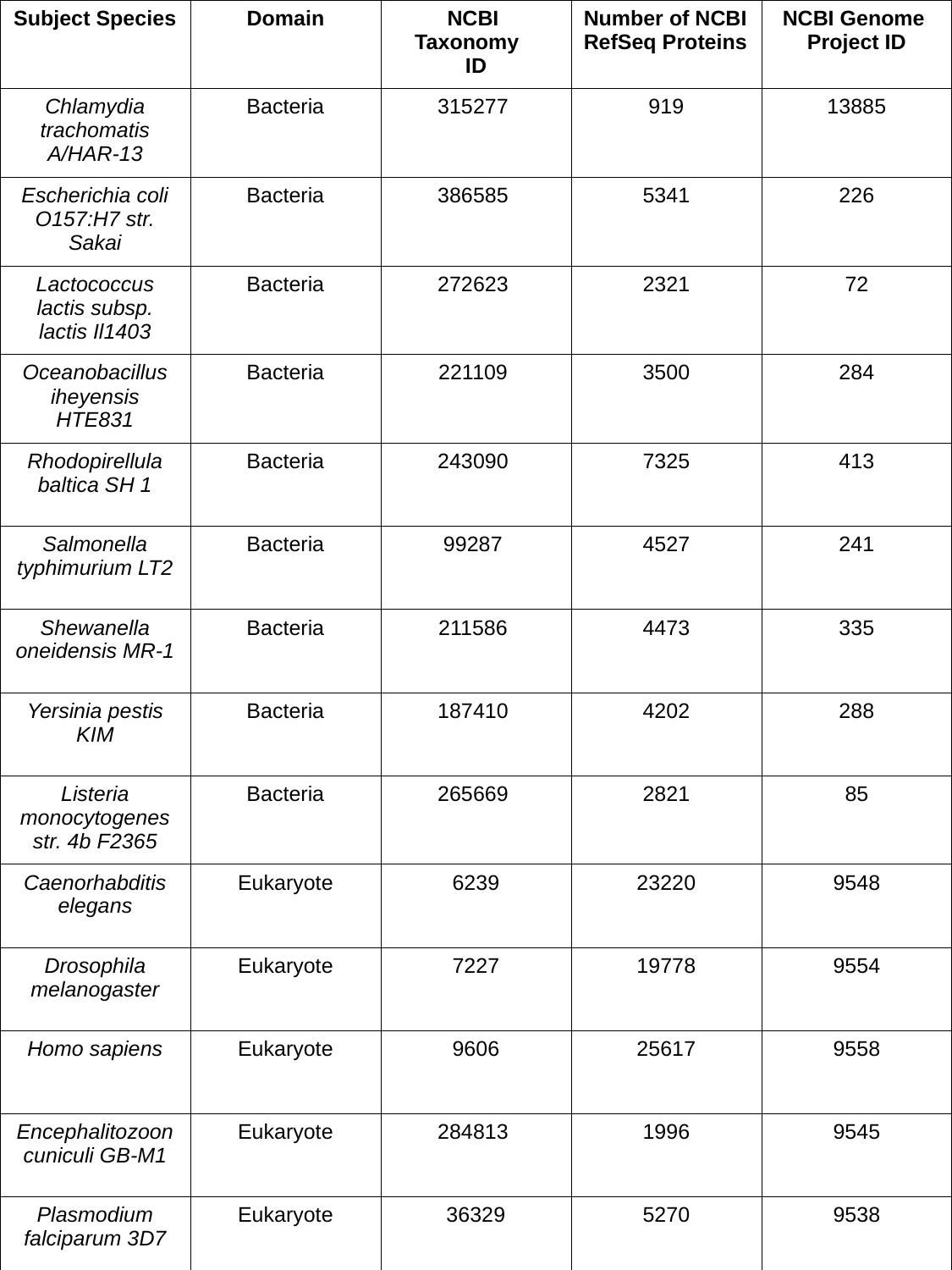

| Subject Species | Domain | NCBI Taxonomy ID | Number of NCBI RefSeq Proteins | NCBI Genome Project ID |
| --- | --- | --- | --- | --- |
| Chlamydia trachomatis A/HAR-13 | Bacteria | 315277 | 919 | 13885 |
| Escherichia coli O157:H7 str. Sakai | Bacteria | 386585 | 5341 | 226 |
| Lactococcus lactis subsp. lactis Il1403 | Bacteria | 272623 | 2321 | 72 |
| Oceanobacillus iheyensis HTE831 | Bacteria | 221109 | 3500 | 284 |
| Rhodopirellula baltica SH 1 | Bacteria | 243090 | 7325 | 413 |
| Salmonella typhimurium LT2 | Bacteria | 99287 | 4527 | 241 |
| Shewanella oneidensis MR-1 | Bacteria | 211586 | 4473 | 335 |
| Yersinia pestis KIM | Bacteria | 187410 | 4202 | 288 |
| Listeria monocytogenes str. 4b F2365 | Bacteria | 265669 | 2821 | 85 |
| Caenorhabditis elegans | Eukaryote | 6239 | 23220 | 9548 |
| Drosophila melanogaster | Eukaryote | 7227 | 19778 | 9554 |
| Homo sapiens | Eukaryote | 9606 | 25617 | 9558 |
| Encephalitozoon cuniculi GB-M1 | Eukaryote | 284813 | 1996 | 9545 |
| Plasmodium falciparum 3D7 | Eukaryote | 36329 | 5270 | 9538 |
