# Supplementary figures and images for: Relationship between insertion/deletion (indel) frequency of proteins and essentiality
Source: BMC Bioinformatics. 2007 Jun 28;8:227. doi: 10.1186/1471-2105-8-227 (PMC1925122; doi:10.1186/1471-2105-8-227)

## Slide 1
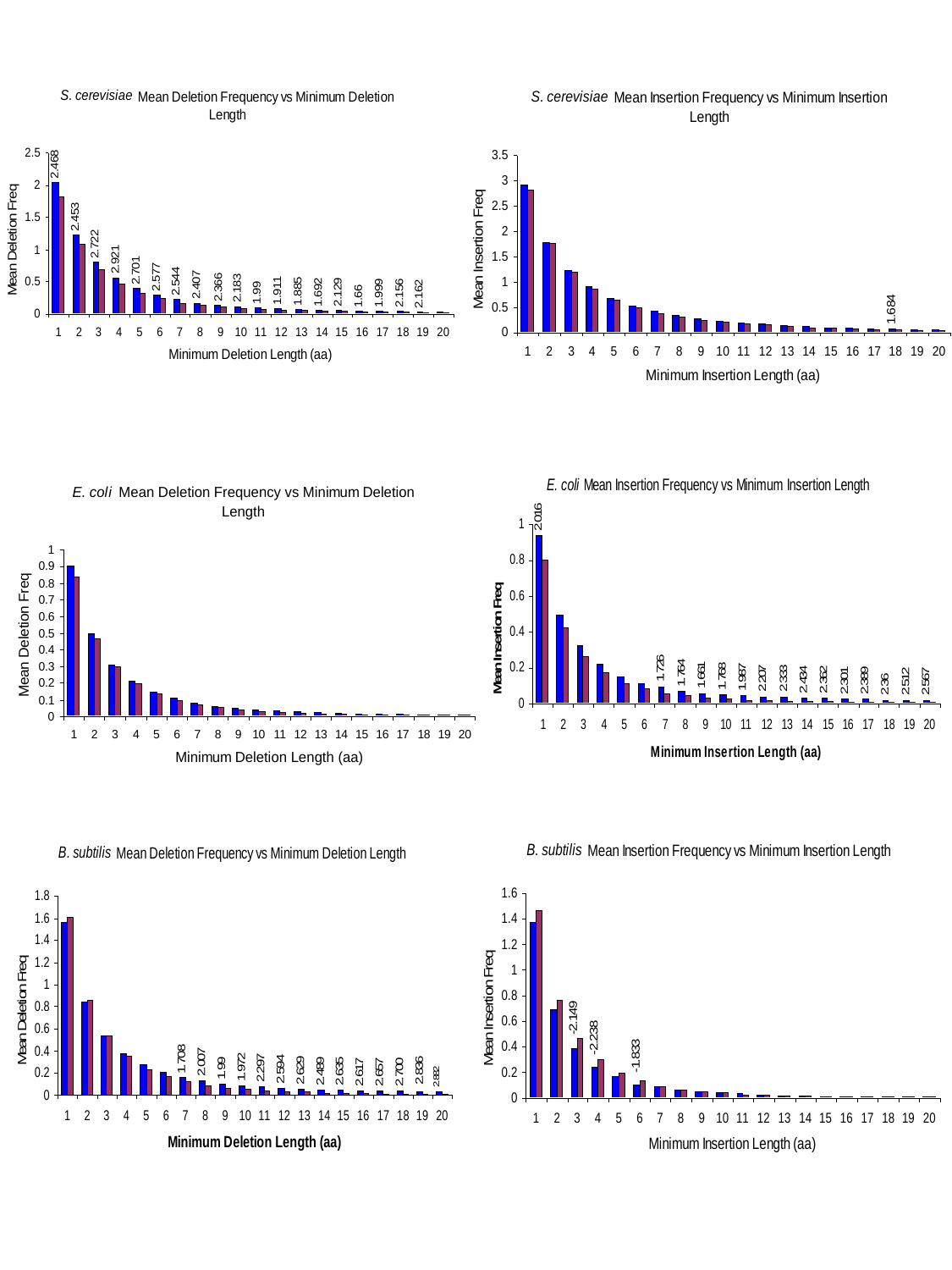

Supplement: Additional File 4 — Mean insertion and deletion frequencies in essential and non-essential proteins plotted against minimum indel length. Mean insertion and deletion frequencies were calculated for essential and non-essential query proteins aligned to the proteins of the 14 randomly chosen subject species. The t-test statistic is shown for the minimum indel lengths that were found significantly more often in essential (blue bars) than non-essential (purple bars) proteins. Significance was set at P < 0.05. Note that no such difference was observed in deletions within E. coli proteins. Also note that insertions of minimum length three, four, and six amino acids were found more frequently in non-essential than essential proteins of B. subtilis. See text for discussion. [file 1471-2105-8-227-S4.ppt]

## Slide 1
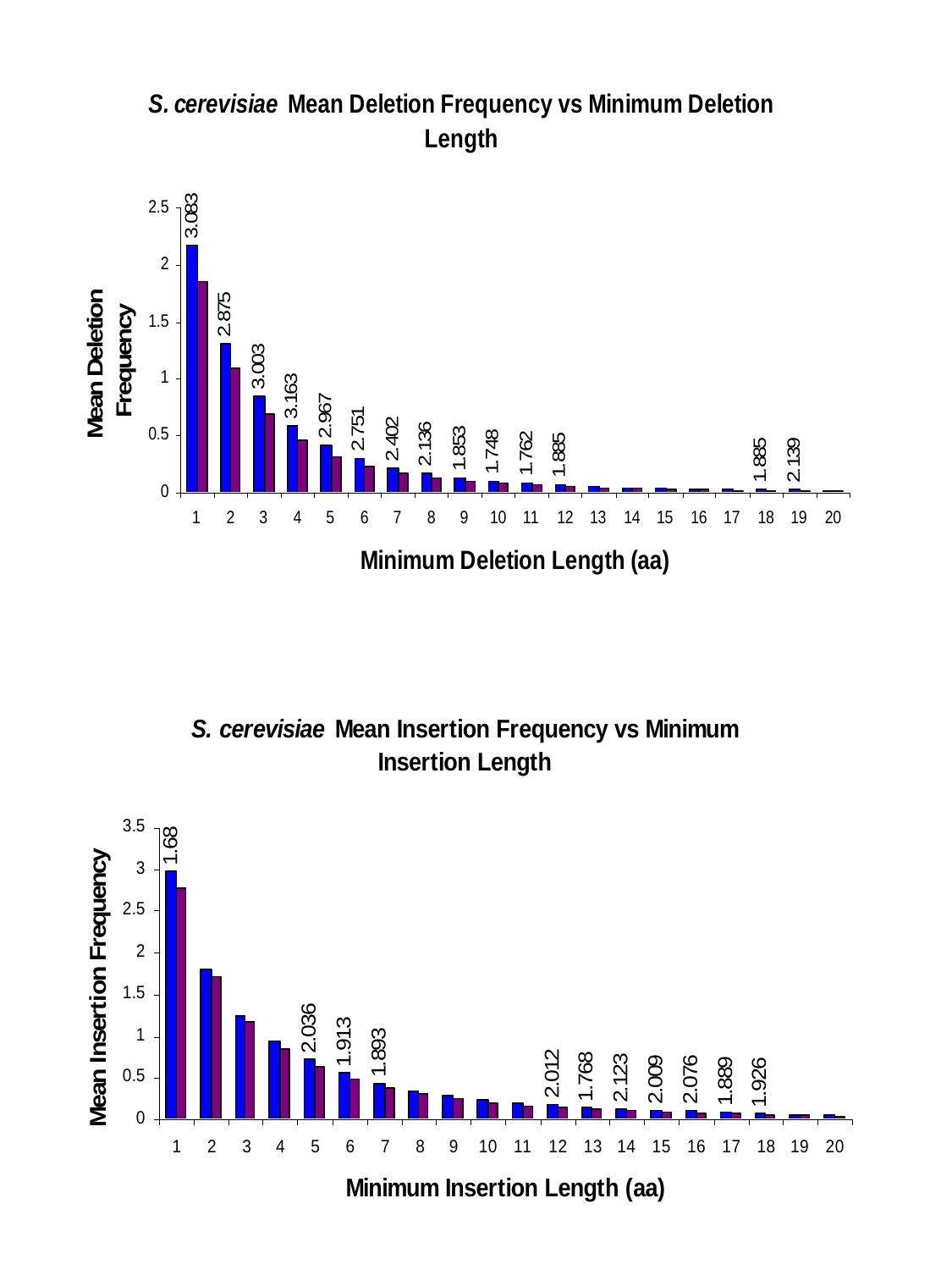

Supplement: Additional File 5 — Mean indel frequency calculated with curated eukaryote proteins. Mean indel frequencies were calculated for the curated S. cerevisiae essential and non-essential proteins aligned to the curated proteins of the five randomly chosen subject species. Note that the observed trend in which the mean indel frequency of essential proteins was greater than that of non-essential proteins was also seen with this smaller set of curated proteins, suggesting that the observed trend seen with the proteins from the complete set of subject species was not merely due to sequencing/annotation errors. The t-test statistic is shown for the minimum indel lengths that were found significantly more often in essential (blue bars) than non-essential (purple bars) proteins. Significance was set at P < 0.05. See text for discussion. [file 1471-2105-8-227-S5.ppt]
